# Artificial intelligence: transforming cardiovascular healthcare in Africa

**DOI:** 10.1186/s43044-024-00551-w

**Published:** 2024-09-06

**Authors:** Patrick Ashinze, Eniola Akande, Chukwu Bethrand, Eniola Obafemi, Olafisoye-Oragbade Oluwatosin David, Suleiman Nasiru Akobe, Ndubuisi Onyinyechukwu Joyce, Obidiegwu Jonathan Izuchukwu, Ngozi Peace Okoro

**Affiliations:** 1 Faculty of Clinical Sciences, University of Ilorin, Ilorin, Nigeria; 2 Chukwuemeka Odumegwu Ojukwu University Teaching Hospital, Anambra, Nigeria; 3 Faculty of Clinical Sciences, University of Nigeria, Nsukka, Nigeria; 4 Department of Medicine, David Umahi Federal University Teaching Hospital, Uburu, Ebonyi State Nigeria

**Keywords:** Artificial Intelligence, Cardiovascular diseases, Africa

## Abstract

**Background:**

Cardiovascular diseases (CVDs), a significant global health concern, are responsible for 13% of all deaths particularly in Africa, where they contribute substantially to the global disease burden, taking several millions of lives globally and annually. Despite advancements in healthcare, the burden of CVDs continues to rise steadily. This comprehensive review critically examines the intersection of artificial intelligence (AI) and cardiovascular disease (CVD) management in Africa. Drawing on a diverse gamut of scholarly literature and empirical evidence, the review assesses the prevalence, impact, and challenges of CVDs in the African context.

**Main body:**

The review highlights the potential of AI technologies to revolutionize CVD care, offering insights into its applications in diagnosis, treatment optimization, and remote patient monitoring. It explores existing literature sourced from databases like PUBMED, Scopus and Google Scholar about the current state of AI implementation in African healthcare systems, which are majorly resource-constrained, discussing successes, limitations, and future prospects. The work includes the prevalence and impact of CVDs in Africa, noting the significant public health burden and economic implications. Current challenges in addressing CVDs are outlined, focusing on resource constraints, healthcare system challenges, and socioeconomic factors. Our review takes a dive into AI’s role in healthcare, emphasizing its capabilities in disease diagnosis, treatment optimization, and patient monitoring, and presents current applications and case studies of AI in African cardiovascular healthcare. It also addresses the challenges and limitations of implementing AI in this context, such as inadequate infrastructure, lack of high-quality data, and the need for regulatory frameworks.

**Conclusion:**

Our review emphasizes the urgent need for collaborative efforts among policymakers, healthcare providers, and researchers to overcome barriers to AI integration and ensure equitable access to innovative healthcare solutions. By fetching existing research and offering practical recommendations, this review contributes to the academic discourse on AI-driven healthcare interventions in Africa, offering an understanding of the opportunities and challenges in leveraging technology to address pressing public health concerns. It calls for increased research, investment, and collaboration to harness AI’s full potential in transforming cardiovascular healthcare in Africa.

## Background

Cardiovascular diseases (CVDs) are a major concern for global health, as they are one of the leading causes of death across the world, taking about 18 million lives globally and annually [1]. Cardiovascular diseases are diseases affecting the heart, its chambers and blood vessels, and can lead to a range of complications such as myocardial infarction, cerebrovascular diseases, rheumatic heart disease, valvular diseases, hypertension and heart failure [1]. Sadly, despite health interventions, policies and medical technology, the worldwide burden of CVD has continued to rise steadily [1].

Nevertheless, owing to the recent advancement of technology, artificial intelligence (AI) has been extensively employed in accurately diagnosing and predicting the prognosis of a multitude of illnesses. Artificial intelligence in medicine is the use of machine learning models to help process medical data and give medical professionals important insights, improving health outcomes and patient experiences [2]. The application of AI techniques demonstrates great potential for expediting the diagnostic and treatment processes for CVDs [3]. For example, there are AI algorithms used to diagnose cardiomyopathy, heart failure, and atrial fibrillation [4, 5]. This is especially important as early diagnosis helps in instituting the right treatment of CVDs and significantly slows the progression to advanced diseases and improves overall outcomes. AI can also be used for real-time disease counseling, medications reminders, and remote follow-ups [5].

Although the potential of AI in cardiovascular disease management for Africa is promising, there may be specific obstacles to its implementation in Africa. For example, the limited access to healthcare facilities and the barest minimum medical facilities and technology, and the lack of adequate and precise data on CVDs in Africa. Overall, while there are challenges to implementing AI for CVDs management in Africa, the potential benefits make it an important area of focus for researchers and policy Makers. In this review, we introduce the growing significance of AI in healthcare, particularly in addressing CVDs.

## Main text

### Prevalence and impact of cardiovascular diseases in Africa

Cardiovascular diseases are an important cause of morbidity and mortality globally. In Africa, CVDs are the largest contributor to the total non-communicable disease (NCD) burden and are responsible for approximately 13% of all deaths and 37% of all NCDs deaths [6]. In Africa, the prevalence of CVDs is on the rise, with about 18 million people living with the condition [7]. Generally, more people die annually from CVDs than from any other cause, with the surge in deaths occurring in low-income countries, i.e., Sub-Saharan African nations [8]. Sub-Saharan Africa is experiencing an epidemic of CVDs. This is attributable to the rapid urbanization and adoption of western lifestyles [9, 10]. This increase is predominantly driven by increased rates of hypertension, smoking, and obesity, [11] with studies showing that mortality by CVD is projected to be more than 23 million by the year 2030 [12]. The impact of CVDs on public health, healthcare systems and economics in Africa can never be overstated. CVDs pose a considerable public health concern as they result in untimely death, disability, diminished work productivity, and high expenses for patients, families and the healthcare sector. In many cases, patients with CVDs must pay out-of-pocket for their medical expenses since health insurance coverage is limited or nonexistent. This can lead to catastrophic health expenditures that push families below the poverty line. Evidence has shown that maintaining patients on guideline-directed medical therapy is challenging, with patients frequently defaulting in treatment, requesting for cheaper options and being lost to follow-up due to financial constraints [13]. This has led to suboptimal treatment, faulty treatment, and increased CVD-related hospitalization in Africa.

### Current challenges in addressing cardiovascular diseases in Africa

The diagnosis, prevention and management of cardiovascular diseases (CVD) in Africa faces a number of multifaceted challenges that stem from the intersection of resource constraints, socioeconomic factors, healthcare system challenges, limited data and research, and political and governance issues [14, 15]. These factors negatively impact the health seeking behavior of Africans and also cripple healthcare delivery significantly.

Poverty and poor socioeconomic background alongside poor health education measures play a crucial role in the health seeking behavior of Africans, and as a result of this, basic healthcare and screening thus becomes a luxury for many as there is little to no sustainable health insurance policy in the vast majority of African countries.

Africa has a huge shortfall in healthcare facilities and personnel, which is further aggravated by the low percentage of GDP invested in healthcare [16]. While infectious diseases seem to be the focus of the government, non-communicable diseases (NCDs), largely including CVDs, are relegated to the background leading to an unabated increase in their prevalence, morbidity and mortality [16]. Scarcity of health data and dearth of innovative research in the field of cardiovascular health also poses a great challenge in the diagnosis and prevention of these ailments.

Finally, there is a deficiency of cost-effective, integrated and evidenced-based approaches for CVD prevention targeted at whole populations and supported by effective policies and government commitment [17].

There are current concerted efforts in some parts of Africa in the establishment of specialist cardiovascular centers, healthcare insurance coverage, universal health education, training and retraining of primary care givers in cardiovascular health. These measures are however still fraught with deep rooted challenges including ease of access, poor technological advancement and coverage, exodus of trained professionals and government apathy.

### Artificial intelligence in healthcare

In its most basic form, artificial intelligence is described as a sophisticated array of computer processes that simulate human intelligence and are capable of carrying out complex tasks that would otherwise have required a human mind [18]. Artificial intelligence is not a singular technology, but rather a collection of several. Relevant examples include machine learning (ML), a statistical technique for fitting models to data and to ‘learn’ by training models with data [18, 19]. Deep learning, a subset of machine learning that involves greater volumes of data, training times, and layers of ML algorithms to produce neural networks capable of more complex tasks. Others are, natural language processing systems, rule-based expert systems and physical robots with predetermined automated tasks [18].

In healthcare, AI can help clinicians diagnose disease and optimize treatment processes. After being applied to traditional medical procedures, it can also reduce the rate of misdiagnosis and improve diagnostic efficiency. Then, with the advent of deep learning, AI has the ability to recognize medical images and provide clinicians with more reliable imaging diagnostic information. By using big data analysis (analyzing extremely large data sets that are difficult to analyze by traditional data processing methods) [19], AI algorithms can often provide more accurate results for patient prediction. It can also help support drug research and improve the efficiency of new drug development. Finally, the combination of artificial intelligence and surgical robots will improve the accuracy of many complex and difficult operations [20].

At present, AI technologies have been applied in cardiovascular medicine including precision medicine, clinical prediction, cardiac imaging analysis and intelligent robots. AI can be initially applied for remote follow-ups, medication reminders, real-time disease counseling and early identification and warnings of symptoms, especially in low-income countries. At the same time, from the perspective of clinicians, AI can help collect voice information (such as medical history), connect electronic medical records systems and reduce the workload of clinicians [21], bridging the gap between huge patient loads, physician scarcity, and universal healthcare access as relates to cardiovascular disease (CVD).

### Current applications of artificial intelligence for cardiovascular diseases in Africa

AI has been employed in managing and sequencing healthcare datasets in Morocco, reading genomes in South Africa, tracking tuberculosis surveillance in Mozambique, analyzing medical images in Ghana and tracking the progression of COVID-19 in Ethiopia [21].

Even more, the benefits of AI tools worldwide in cardiovascular health have long been lauded, and multiple tools and techniques are being developed and rigorously tested around the world; however, there has been a significant gap in its research and application in Africa. While other disease conditions like malaria in Senegal, Nigeria, Cameroon, and DRC, pediatric HIV in Mozambique and South Africa, pulmonary TB in Zambia and South Africa, depression in Kenya, diarrhea among children under five years in Mali, and Zimbabwe, Lassa and viral hemorrhagic fevers in West Africa, and HIV/AIDS in South Africa, have had predictive models developed to guide treatment and response [22], cardiovascular disease (CVD) has lagged behind.

One of the few examples involves an external validation study in Uganda where the mayo clinic AI-ECG showed good accuracy, sensitivity and specificity to detect left ventricular systolic dysfunction (LVSD) in patients seen in a clinical setting [23]. This tool may facilitate the identification of people at a high risk for LVSD in settings with low resources; however, wider implementation is yet to be seen.

Another example is in Bamako, Mali, where Point G hospital, in collaboration with Healthtech Mali, has introduced AI systems into its cardiology department to enhance diagnostics and treatments for patients with heart conditions [24]. Through this collaboration, cardiologists have access to advanced AI tools, facilitating the interpretation of medical data and informed decision-making [24].

The Mayo clinic AI-ECG tool is a convolutional neural network trained on the paired 12 lead ECG and echocardiogram data of 44,959 patients to identify patients with ventricular dysfunction [25]. Unfortunately, few details are available about the exact nature of AI systems in places like Bamako and the Sub-Saharan at large.

#### Challenges and limitations of implementing AI in African context

The benefits of implementing AI in cardiovascular disease prevention and care in Africa are obvious but introducing and implementing them poses challenges, including inadequate infrastructure and data processing and storage facilities, as well as understanding, trust, and acceptance of such applications [26]. Socioeconomic disparities also play a crucial role, as significant disparities in healthcare access and quality exist across different socioeconomic groups in Africa, and the limited healthcare workforce and expertise in AI and data science in many African countries further exacerbate these challenges [26, 27].

Many regions lack the necessary electricity supply and internet access for the reliable implementation of large-scale AI projects. Moreover, access to high-quality locally generated data sets is required to improve on the available tools; however, there is a significant lack of digitized health data and organized collection and collation, so most data sets are collected and stored outside Africa, which limits their use in tackling healthcare issues specific for African people [27]. Also, AI research is incredibly expensive and time-consuming, and while CVDs are of public health concern, more pressing issues like infectious diseases (HIV, TB), neglected tropical diseases and malaria receive far more attention and by default, significantly more funding [26, 27].

Furthermore, data privacy concerns constitute a significant barrier, as many African countries lack robust data protection laws and regulations, and cultural and social sensitivities around sharing personal health information persist [27]. Additionally, limited access to reliable electricity, internet, and computer hardware, as well as inadequate healthcare infrastructure, hinders the adoption of AI in healthcare [27].

The need to ensure patient safety and data privacy also comes to the fore when handling large amounts of confidential patient data and significant research and regulation still remains lacking in this area, further limiting the speed of adoption and scaling.

#### Reducing CVDs in Africa: opportunities and prospects

Advancements in technology come with improvements in various aspects of human life, and in healthcare, these innovations can go a long way in preventing diseases, promoting health, and preserving life. With the increasing burden of cardiovascular disease in Africa, which has emerged as a significant public health concern, it has become necessary to not only address these problems, but also seek out more lasting and effective solutions for better health outcomes. Bulto et al. noted that Africa is facing a double burden of disease where patients are suffering from non-communicable diseases (NCDs) such as coronary disease, along with the burden of infectious diseases and nutritional problems [28]. In the past decades, ~ 80% of CVD deaths occurred in developing countries. In 2019, > 1 million deaths were attributable to CVD in sub-Saharan Africa, which constituted 5.4% of all global CVD-related deaths and 13% of all deaths in Africa [28]. Therefore, tackling this problem in Africa would drastically improve global health status.

There is much room for AI integration in Africa’s healthcare system, particularly in the cardiovascular sector. AI can, among other things, provide adequate complementary efforts to physicians to deal with the increased burden of cardiovascular diseases in Africa. Its incorporation will enable faster diagnosis, early detection of disease conditions, proper monitoring, and many more. This will allow the limited hands in the African healthcare sector to be better equipped to handle these problems. Africa undoubtedly has unique challenges that may limit the benefits of AI integration in her healthcare sector. However, we must collectively rise above these challenges and maximize this technology. A study suggests involving local partners, including policymakers, regulatory bodies, healthcare providers, and patients, from the early stages of innovation development is critical [29]. Their input and feedback can guide the design and implementation of digital or other solutions that align with local needs and priorities [29]. Through proper training with these AI tools, healthcare providers can move from outdated healthcare delivery models to modern and more effective methods.

Echocardiography is central to the assessment and management of all cardiac diseases [30]. It remains the predominant imaging modality for the timely and cost-effective evaluation of cardiac structure and function. It is also a major area where more research into AI integration can be made. The strength and prospect of echocardiography AI research is in the identification of subtle or unrecognized imaging features that may represent subclinical disease or indicate patient prognosis [31].

The prospect is bright; however, reducing cardiovascular disease (CVD) in Africa requires a comprehensive approach that combines public health initiatives, enhanced healthcare infrastructure, and the integration of AI technology. Public health education should be reimagined to resonate with diverse communities, emphasizing the major risk factors for CVDs. Strengthening primary healthcare and deploying mobile clinics can bridge gaps in access, especially in rural areas, while AI can improve early detection, diagnostics, and personalized treatment. National policies focused on CVD prevention, alongside investments in AI, research, and international collaborations, are essential for effectively reducing the CVD burden across the continent.

### Recommendations

To reiterate, enhancing healthcare infrastructure in Africa requires a concerted effort from governments, stakeholders, and international organizations to build and upgrade facilities, provide essential diagnostic tools, and ensure consistent electricity and internet access to support AI implementation in the assessment, diagnosis, prognosis and management of cardiovascular diseases—inherited and acquired.

Investment in training and retaining cardiologists, cardiovascular surgeons, interventional specialists, nurses, biomedical engineers and allied healthcare professionals is crucial, alongside incentives to curb the brain drain in the continent and attract talent to underserved areas. Expanding health insurance coverage is essential to make healthcare more affordable and accessible.

Comprehensive AI strategies should be developed by fostering local and international collaborations to create AI tools tailored to African needs. Training healthcare professionals in AI technologies and integrating AI-focused curricula into medical and technical schools are vital steps. Strengthening data infrastructure will enhance the quality and availability of data necessary for effective AI applications.

Public health efforts should focus on preventive measures through large-scale awareness campaigns, community health programs for early detection and monitoring of CVD risk factors, and promoting healthy lifestyle changes through policy interventions. Strong health policies that prioritize CVD prevention and management, coupled with clear regulatory frameworks for AI in healthcare, are essential to ensure the safe and effective implementation of AI technologies. Engaging stakeholders, including local communities, healthcare providers, and patients, will ensure culturally appropriate and widely accepted solutions.

International partnerships and funding are critical, with support from donors, NGOs, and private sector partners to finance CVD initiatives and AI integration. Collaborative research will allow for the sharing of knowledge and resources. For disease management, AI can be used for remote monitoring of CVD patients, providing real-time data and improving medication adherence, while telemedicine services can expand access to care, particularly in rural areas.

Encouraging local innovation by supporting startups and establishing innovation hubs focused on healthcare technology and AI will foster a culture of entrepreneurship and drive the development of local solutions tailored to the region’s specific healthcare challenges.

## Conclusion

Considering the growing burden of cardiovascular diseases in Africa, the emergence of new technology to deal with this problem cannot be overemphasized. These diseases have led to increased disability, decreased work productivity, heavy financial burdens, and increased mortality in the African population. It highlights the need for a better approach to dealing with this problem.

The introduction of AI technology into medicine has helped produce better health outcomes across the world. While the rest of the world advances with this technology, Africa seems to lag. There is a lot we stand to gain from the integration of AI technology in the management of cardiovascular diseases. Its incorporation can lead to earlier and faster diagnoses, better follow-up measures, and better health outcomes. We have barely scratched the surface as a continent, and this is one area where we need more research, investment, and collaboration to rise above the challenges. By channeling our resources into this area, we can reduce the burden of non-communicable diseases in Africa.

The current limitations to AI integration in cardiovascular healthcare pale compared to its overwhelming benefits. Therefore, all hands should be on deck to ensure we make the most of this technological improvement to provide better healthcare services in Africa.

## Data Availability

Not applicable.

## Abbreviations

AI: Artificial Intelligence
CVD: Cardiovascular Disease
CVDs: Cardiovascular Diseases
ML: Machine Learning
NCD: Non-communicable Disease
NCDs: Non-communicable Diseases
LVSD: Left Ventricular Systolic Dysfunction
DRC: Democratic Republic of the Congo
HIV: Human Immunodeficiency Virus
TB: Tuberculosis
